# MicroRNA-30a-3p Influences Milk Fat Metabolism by Targeting *PTEN* in Mammary Epithelial Cells of Sheep

**DOI:** 10.3390/ani15081180

**Published:** 2025-04-20

**Authors:** Yamin Guo, Xinmiao Wu, Huimin Zhen, Yuxin Feng, Mingna Li, Chunyan Ren, Jiqing Wang, Zhiyun Hao

**Affiliations:** 1 Gansu Key Laboratory of Herbivorous Animal Biotechnology, College of Animal Science and Technology, Gansu Agricultural University, Lanzhou 730070, China; guoym2025@163.com (Y.G.); wuxinmiao2020@163.com (X.W.); zhenhm@st.gsau.edu.cn (H.Z.); 17789574669@163.com (Y.F.); limn@gsau.edu.cn (M.L.); renyaya86@126.com (C.R.); 2 Department of Bioengineering, Linxia Modern Vocational College, Linxia 731199, China

**Keywords:** microRNA-30a-3p, MECs, sheep, mammary gland, lactation

## Abstract

Our previous investigations identified miR-30a-3p as a differentially expressed miRNA in ovine mammary tissue across sheep breeds with distinct lactation performance and different physiological stages. However, its regulatory mechanisms controlling mammary gland development and lactation remain unexplored. In this study, the effect of miR-30a-3p on the proliferation of ovine mammary epithelial cells (MECs) and the target genes of miR-30a-3p were investigated. The regulatory effects of miR-30a-3p on the expression of the target genes and the content of triglycerides in ovine MECs were also analyzed. The transfection of miR-30a-3p mimic was found to promote cell viability and the number of proliferated ovine MECs using CCK8 and Edu assays. On the contrary, the miR-30a-3p inhibitor showed the opposite results with the miR-30a-3p mimic. These results suggest that miR-30a-3p promotes the proliferation of ovine MECs. The dual luciferase assay revealed that Phosphatase and Tensin Homolog (*PTEN*) can be targeted with miR-30a-3p. The transfection of miR-30a-3p mimic into ovine MECs resulted in a significant decrease in expression levels of the target *PTEN* genes. However, it promotes five milk fat synthesis marker genes, *ACSL4*, *AKT*, *SREBP1*, *mTOR*, and *LPL*, and promotes the content of triglycerides. The opposite effect of miR-30a-3p inhibitor with mimic on the expression levels of these genes and the level of triglycerides was also observed. This is the first study to reveal the biological mechanisms by which miR-30a-3p promotes milk fat synthesis by targeting *PTEN* in sheep.

## Simple Summary

Previous research identified miR-30a-3p as differentially expressed in ovine mammary glands between breeds with varying lactation performance and across lactation stages. This study found that overexpression of miR-30a-3p significantly downregulated *PTEN* expression while upregulating milk fat synthesis marker genes (*ACSL4*, *AKT*, *SREBP1*, *mTOR*, *LPL*) and increasing triglyceride content in ovine mammary epithelial cells (MECs). Conversely, miR-30a-3p inhibitor markedly elevated *PTEN* expression, reduced these marker genes, and decreased triglycerides. These findings demonstrate that miR-30a-3p promotes ovine MECs proliferation and milk fat synthesis by targeting *PTEN*, which may modulate key lipid synthesis pathways.

## 1. Introduction

Sheep milk, characterized by its abundant high-quality proteins, calcium, and natural vitamins, exhibits superior digestibility, enhanced nutrient absorption efficiency, and reduced allergenicity, thereby serving as an exceptional natural nutritional reservoir for infants, immunologically sensitive populations, and health-conscious individuals [1,2,3,4]. It also acts both as a critical reservoir of essential nutrients and a dynamic conduit for bioactive molecular signaling that orchestrates offspring development and physiological programming [5,6]. For lambs, the demand for colostrum is particularly critical, with an immediate requirement of 50 mL per 1 kg of birthweight [7]. When ewes give birth to multiple lambs, insufficient milk production can lead to stunted growth or even mortality in the lambs [8,9]. The risk increases with lambs, as the nutritional demands of multiple lambs often exceed the ewe’s milk production capacity [7]. Adequate milk supply depends on several factors, including ewe breed, management, environment, nutrition, and udder development [5,7]. Milk is secreted and synthesized by mammary epithelial cells in the mammary parenchyma and involves the processes of mammary epithelial cell differentiation, synthesis and secretion of milk components, and maintenance of mammary homeostasis [5,10,11]. Any factor that interferes with these processes will interfere with milk secretion or even lead to lactational cessation [10,12]. Recent studies have revealed that lactation performance is regulated by a complex network of genetic and molecular factors, including coding and non-coding genes [11,13].

MicroRNAs (miRNAs) are small non-coding RNAs that post-transcriptionally regulate gene expression by targeting specific mRNAs for degradation or translational repression [14,15]. Over the past decade, miRNAs have emerged as key regulators in mammary gland biology, influencing diverse processes such as cell proliferation, differentiation, and apoptosis [11,16,17,18,19]. For example, Let-7g-5p is downregulated in the mammary glands of mice during pregnancy, where it plays a crucial role in regulating mammary cell differentiation and function by targeting protein kinase C alpha [20]. Additionally, the same study revealed that miR-142-3p is decreased in the mammary glands of lactating mice, thereby modulating milk synthesis and the structure of prolactin receptor-mediated signaling pathways [21]. However, most relevant studies on the effects of miRNAs on lactation performance have focused on humans, dairy cows, and dairy goats, and even less on sheep [11]. This is not conducive to our understanding of the regulatory mechanisms of lactation in sheep. Recent studies in sheep have further highlighted the involvement of miRNAs in lipid synthesis at the post-transcriptional level [17,19,22,23,24]. For example, miR-200c has been shown to promote triglyceride synthesis in ovine MECs by increasing the lipid synthesis-related gene expression by targeting *PANK3* [19]. More importantly, miRNAs can regulate multiple target genes in an organism and play different roles in different tissues. We, therefore, need to explore more functions of miRNAs to explain the complexity of lactation traits [14,15].

In our previous study, we found that miR-30a-3p expression was significantly upregulated in lactating sheep mammary glands compared to non-lactating sheep mammary glands [24]. As a member of the miR-30 family, miR-30a-3p has been implicated in regulating cell cycle progression, metabolic homeostasis, and cancer pathogenesis. A study by Tian et al. (2023) demonstrated that miR-30a-3p inhibits autophagy and alters mammary structure during the involution of mouse mammary glands, highlighting its role in mammary remodeling [25]. Notably, its family member miR-30a-5p is significantly downregulated in non-lactating compared to lactating mammary glands [26]. These results imply that miR-30a-3p might play a regulatory role in modulating mammary tissue growth and milk production efficiency within the ovine species. However, the function of miR-30a-3p in mammary gland development and lactation in sheep has not been reported.

Therefore, in this study, we systematically detected miR-30a-3p expression levels in eight different tissues in sheep, including the mammary gland, and in the mammary gland at six key developmental stages using quantitative real-time PCR (RT-qPCR). We also investigated the effect of miR-30a-3p on the proliferation of ovine mammary epithelial cells (MECs) and characterized the target genes for miR-30a-3p using Edu, CCK8, RT-qPCR, and Western blotting. We also assessed the effects of miR-30a-3p on the expression of the target genes and the content of triglycerides in the ovine MECs. Finally, we will reveal the expression profile of miR-30a-3p at different developmental periods of the mammary gland and in different tissues and further elucidate its effects on ovine MECs. Our findings may provide critical insights into potential therapeutic targets for enhancing milk production and maintaining mammary gland health in domestic animals.

## 2. Materials and Methods

### 2.1. Ethics Statement

All experimental procedures were conducted in strict compliance with international guidelines for animal research and were approved by the Institutional Animal Care and Use Committee (IACUC) of Gansu Agricultural University (Approval No. GSAU-Eth-AST-2024-006). This approval confirms adherence to ethical standards for the humane treatment and use of laboratory animals.

### 2.2. Collection of Ovine Tissue Samples

Six healthy three-year-old small-tailed Han ewes at fourth parity were selected from Kangyuan Sheep Farm (Huining County, China). All animals were maintained under a uniform feeding regimen, and the ewes exhibited homogeneous body weight (mean ± SD: 53.75 ± 1.87 kg). All ewes were artificially inseminated by simultaneous estrus, and successful conception was determined by a veterinary ultrasound instrument. Then, mammary parenchyma tissues were collected from three ewes (*n* = 3) across six distinct physiological stages, including non-pregnant (30 days after the cessation of lactation), pre-pregnancy (45 days post-conception), late pregnancy (140 days post-conception), pre-lactation (15 days postpartum), mid-lactation (30 days postpartum), and late lactation (60 days postpartum), according to the surgical method [27]. In detail, ewes were anesthetized under aseptic conditions, the udder skin was incised after disinfection with iodine vapor, and a sample of mammary parenchymal tissue was completely excised. The samples were immediately placed in liquid nitrogen; the muscularis propria was sutured to the skin in layers, and antibiotics were injected after surgery to prevent infection. Meanwhile, three mid-lactation ewes were euthanized for additional tissue collection (mammary gland, longissimus dorsi muscle, heart, liver, kidney, spleen, lung, and ovary) to analyze miR-30a-3p expression patterns.

### 2.3. Isolation and Culture of Ovine MECs

Primary ovine MECs were isolated from the pre-lactation mammary gland using our previously described methodology [19]. In detail, mammary parenchyma was washed thrice with PBS (0.01 M, pH 7.4) containing 500 IU/mL penicillin/streptomycin and sectioned into 1 mm^3^ explants. These explants were evenly spaced (1 cm intervals) in culture flasks and precultured in an inverted orientation at 37 °C for 3 h. The primary culture was subsequently established in DMEM/F12 medium supplemented with 10% fetal bovine serum (FBS) under 5% CO_2_ at 37 °C, with medium replenished every 48 h. Distinct cellular migration from explant margins was typically observed on day 7 of the culture. Then, fibroblast contamination was eliminated through differential trypsinization [19]. Purified ovine MECs were cultured in DMEM/F12 medium (Gibco, Grand Island, NY, USA) containing 5 μg/mL 17-β-estradiol, 10 μL/mL insulin–transferrin–selenium complex, 5 μg/mL hydrocortisone, 10 ng/mL EGF, and 10% FBS, with incubation at 37 °C in 5% CO_2_. Finally, prolactin (2 μg/mL) was added at the beginning of the experiment to induce sheep MECs to start differentiating.

### 2.4. Cell Viability, Proliferation, and Triglyceride Content Detection

The ovine MECs were seeded in a 48-well plate at 2 × 10^5^ cells/well and transiently transfected when cells were at 70% confluence using 50 nmol/L miR-30a-3p mimics or 100 nmol/L inhibitor, with respective negative controls (RiboBio, Guangzhou, China). All transfections were performed in nine copies using INVI™ transfection reagent (Invigentech, Irvine, CA, USA). Then, the viability and proliferation of ovine MECs were determined using CCK8 and EDU Kits according to our previously described methodology [19]. Then, the ovine MECs nucleus was stained with Hoechst 33258 (Solarbio, Beijing, China); the proliferation of ovine MECs was labeled with Edu using the Cell-Light™ Edu Apollo^®^ 567 In Vitro Imaging Kit (RiboBio, Guangzhou , China) according to the instructions (*n* = 3). Finally, the results were visualized using a biological microscope IX73 (Olympus, Tokyo, Japan), and the data were analyzed using Image J v.1.8.0 software (NIH, Bethesda, MD, USA). Meanwhile, after 48 h of transfection, the intracellular triglyceride (TAG) levels were quantified using a cell triglyceride assay kit (Solarbio, Beijing, China), following the manufacturer’s instructions. Three replications of each of the above experiments were performed. The triglyceride content was normalized according to the expression of the corresponding negative controls.

### 2.5. Target Gene Prediction and Validation

The binding sites of miR-30a-3p and target genes were identified using miRanda 3.3a, miRDB, and TargetScan 8.0, and then, the predictions from the three kinds of software were overlapped. The Kyoto Encyclopedia of Genes and Genomes (KEGG) pathway of the target genes predicted for miR-30a-3p was analyzed using KOBAS 3.0. The target genes of miR-30a-3p were screened based on the results of the analyses and previous studies [22,23]. To validate miRNA–mRNA interactions, complementary sequences between the mature miRNA seed region and target 3′UTR were used for primer design. Wild-type (WT) and mutant (MUT) luciferase reporter constructs were engineered using NotI/XhoI restriction cloning (RiboBio, Guangzhou, China). For functional testing, HEK 293T cells at 70% confluence were co-transfected with 0.5 ug of either WT or MUT constructs alongside miRNA mimics, with parallel negative controls. Luciferase activity quantification was performed 48 h post-transfection using the Dual-Glo^®^ system (Promega, Madison, WI, USA).

### 2.6. Relative Expression Analysis

The total RNA isolation was performed 48 h post-transfection on ovine MECs and tissue samples using TRIzol™ reagent (Invitrogen, Carlsbad, CA, USA). RNA integrity was assessed spectrophotometrically (NanoDrop™ 2000, Thermo Scientific, Waltham, MA, USA) and electrophoretically (Bioanalyzer 2100, Agilent, Santa Clara, CA, USA). Reverse transcription was conducted with strand-specific protocols: mRNA-derived cDNA was generated using FastKing gDNA Dispelling RT SuperMix (Tiangen Biotech, Beijing, China) with oligo(dT) primers, while small RNAs were reverse-transcribed using the Mir-X™ miRNA First-Strand Synthesis Kit (Takara Bio, Tokyo, Japan). All primers were designed with Primer 5.0 based on sequences retrieved from NCBI (Table S1). Meanwhile, *GAPDH* and *U6* served as reference genes for normalizing mRNA and miRNA expression, respectively [19]. RT-qPCR was performed on the Applied Biosystems QuantStudio^®^6 Flex system (Thermo Lifetech, Bohemia, NY, USA) using SYBR Premix Ex Taq II (Takara, Dalian, China) for mRNA and the miRcute miRNA qPCR Detection Kit (Tiangen, Beijing, China) for miRNAs [15]. Relative gene expression was quantified using the 2^−∆∆Ct^ method [28].

Total cellular proteins were extracted using RIPA lysis buffer and quantified with a BCA kit (Solarbio, Beijing, China). Proteins were separated by 12% SDS-PAGE (Solarbio) and transferred to PVDF membranes (Millipore, Burlington, VT, USA) for 1 h. After blocking with 5% skim milk in TBST for 3 h at room temperature, membranes were incubated overnight at 4 °C with primary antibodies: anti-PTEN (1:200) and anti-actin (1:1000) (both from Proteintech, Wuhan, China). Following three 10-min TBST washes, membranes were incubated with horseradish peroxidase-conjugated secondary antibody (goat anti-rabbit IgG, Proteintech China) at room temperature for 1 h. Protein bands were visualized using a chemiluminescent substrate (Tanon, Shanghai, China) and quantified with ImageJ v.1.8.0.

### 2.7. Statistical Analysis

All experimental data are shown as mean ± SD. Statistical analyses were performed using SPSS 22.0 software (SPSS Inc., Chicago, IL, USA). Inter-group comparisons were conducted with a two-tailed independent Student’s *t*-test, while one-way ANOVA was applied for multi-group comparisons. Data visualization was performed using GraphPad Prism v.8.0.1 (GraphPad, San Diego, CA, USA).

## 3. Results

### 3.1. MiR-30a-3p Is Associated with the Development and Lactation of the Mammary Gland

**Figure 1:**
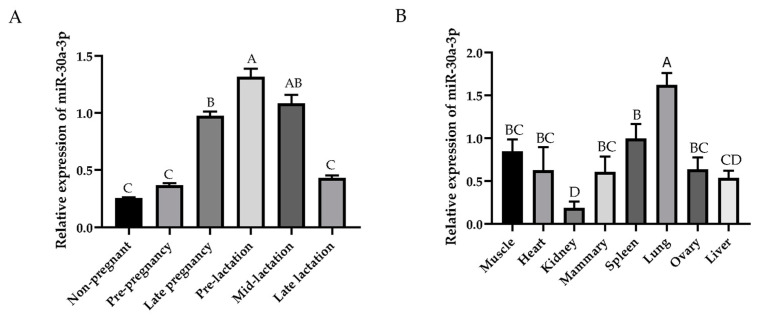
The expression level of miR-30a-3p in mammary gland at different developmental stages (**A**) and in the ovine eight different tissues (**B**). The values are shown as mean ± SD (*n* = 3). Values with different uppercase letters are different at *p* < 0.01.

The expression level of miR-30a-3p exhibits temporal and spatial specificity, with the highest expression level observed in the pre-lactation period of the mammary gland, followed by the mid-lactation period and the lowest expression level observed in the non-pregnancy period (Figure 1A). In detail, the expression level of miR-30a-3p in the pre-lactation period of the mammary gland was significantly higher than that observed in other stages of the mammary gland (*p* < 0.01), with the exception of mid-lactation. The expression levels in the pre-lactation period were 5.20-fold, 3.58-fold, 3.06-fold, 1.35-fold, and 1.26-fold higher than those in the non-pregnancy, pre-pregnancy, late-lactation, late-pregnancy, and mid-lactation stages, respectively.

Meanwhile, the RT-qPCR results indicated that miR-30a-3p was widely expressed in the heart, lung, liver, spleen, ovary, kidney, muscle, and mammary gland. The highest expression of miR-30a-3p was found in the lung, which was highly significant compared to the other tissues (*p* < 0.01). The expression level of miR-30a-3p was higher in mammary gland tissue than in the kidney (*p* < 0.01), but it was not significant in the spleen, ovary, muscle, heart, and liver. The expression level of miR-30a-3p was lowest in the kidney, which was significantly lower than in other tissues except for the liver (*p* < 0.01).

### 3.2. miR-30a-3p Affects the Proliferation of Ovine MECs

**Figure 2:**
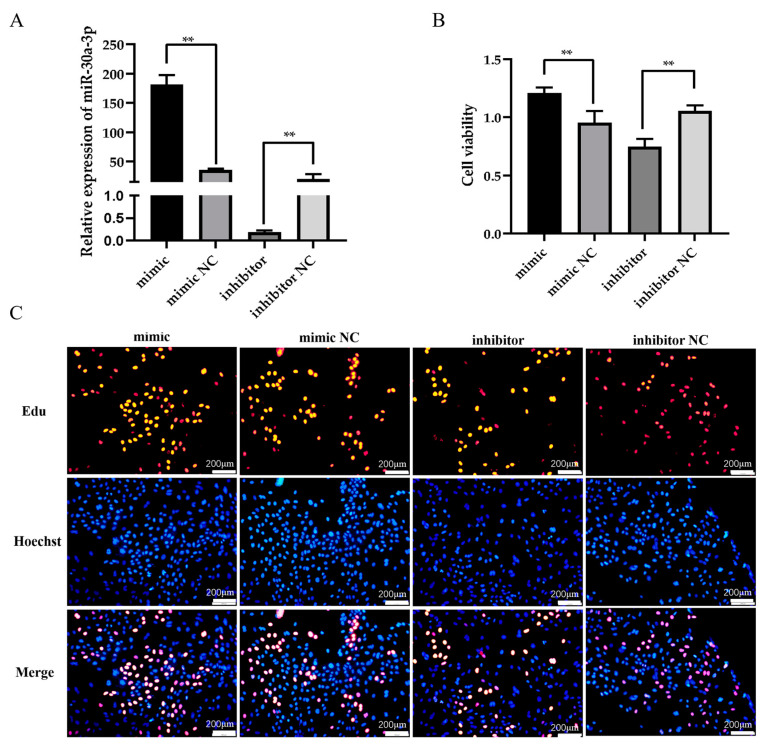
Functional analysis of miR-30a-3p in ovine MECs. (**A**) Transfection efficiency validation of miR-30a-3p modulators (mimic/inhibitor) in ovine MECs. (**B**) The CCK8 assay for detecting the viability of ovine MECs. (**C**) The Edu assay for detecting the proliferation of ovine MECs. The values are shown as mean ± SD (*n* = 3). Blue dots indicate unproliferated cells, others color dots indicate Edu-labeled proliferating cells. ** *p* < 0.01.

The transfection efficacy of miR-30a-3p mimics and inhibitors was detected by RT-qPCR. The results of this study showed a highly significant increase in miR-30a-3p expression levels upon transfection with miR-30a-3p mimic (Figure 2A, *p* < 0.01). In contrast, significant suppression of miR-30a-3p expression was observed when the miR-30a-3p inhibitor was used in ovine MECs (Figure 2A, *p* < 0.01). Furthermore, the CCK8 assay indicated that upregulation of miR-30a-3p significantly increased the viability of ovine MECs, whereas downregulation of miR-30a-3p had an inhibitory effect on cell viability (Figure 2B, *p* < 0.01). Furthermore, the Edu incorporation assay showed that overexpression of miR-30a-3p led to an increase in the number of proliferating cells labeled with Edu, whereas inhibition of miR-30a-3p resulted in a decrease in the proliferation rate of ovine MECs (Figure 2C).

### 3.3. miR-30a-3p Functional Enrichment Analysis and Target Gene Prediction

**Figure 3:**
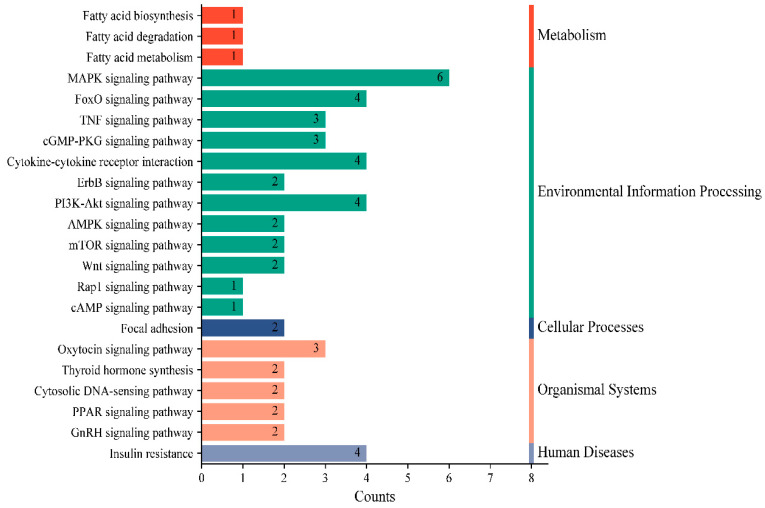
Results of the KEGG pathway annotation of the predicted target genes of miR-30-3p. The X-axis represents the number of genes, and the Y-axis represents the pathway.

A comprehensive prediction using miRanda 3.3a, miRDB, and TargetScan 8.0 identified a total of 86 putative target genes shared by miR-30a-3p. Subsequent KEGG pathway analysis revealed that these target genes were predominantly enriched in 22 metabolic pathways, including fatty acid biosynthesis, degradation, and metabolism (Figure 3). Notably, the target genes were also annotated to several signaling cascades relevant to mammary gland development and lactation processes, specifically the PI3K-Akt, mTOR, focal adhesion, and insulin resistance signaling pathways.

### 3.4. PTEN Is a Direct Target of miR-30a-3p

**Figure 4:**
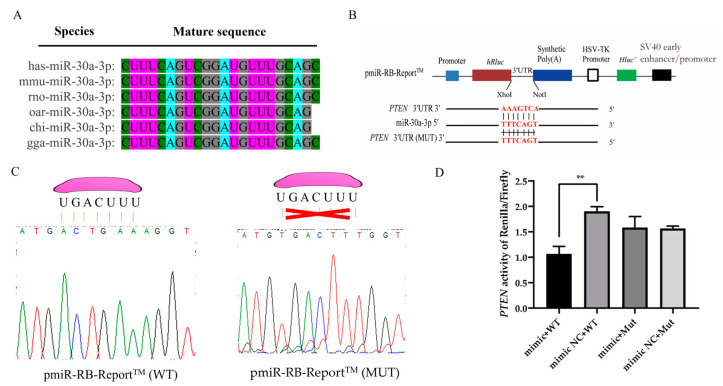
The miR-30a-3p specifically targets the *PTEN* gene in ovine MECs. (**A**) Comparative homology analysis of miR-30a-3p sequences across diverse species underscores its evolutionary conservation. (**B**) Schematic representation of the wild-type and mutant dual-luciferase reporter vector constructs. (**C**) Sanger sequencing confirms the accurate insertion of the miR-30a-3p target sequence (highlighted in pink irregular shape) within the reporter constructs. A red error sign means that the seed sequence and the binding sequence cannot be matched. (**D**) Dual luciferase activity assay results. The values are shown as mean ± SD (*n* = 3). ** *p* < 0.01.

Sequence alignment analysis revealed a high degree of conservation in the mature sequence of miR-30a-3p across diverse species, suggesting its robust evolutionary preservation (Figure 4A). Target prediction analyses indicated that the seed region of miR-30a-3p could form a complete complementarity with the 3′UTR segment of the *PTEN*. To confirm this targeting interaction, a dual-luciferase reporter system was meticulously constructed (Figure 4B). Sanger sequencing further confirmed the presence of the anticipated 3′UTR sequence of the miR-30a-3p target gene in both the wild-type and mutant pmiR-RB-Report™ vectors, marking the successful construction of the reporter system (Figure 4C). Dual-luciferase reporter assays demonstrated that overexpression of miR-30a-3p led to a marked decrease in the Renilla/Firefly luciferase activity ratio, specifically in the PTEN wild-type pmiR-RB-Report™ vector, whereas no significant effect was observed in the PTEN mutant counterpart (Figure 4D). Taken together, these findings provide conclusive evidence that *PTEN* is a direct target gene of miR-30a-3p.

### 3.5. miR-30a-3p Affects the Milk Fat Synthesis Process by Regulating the Expression of PTEN

**Figure 5:**
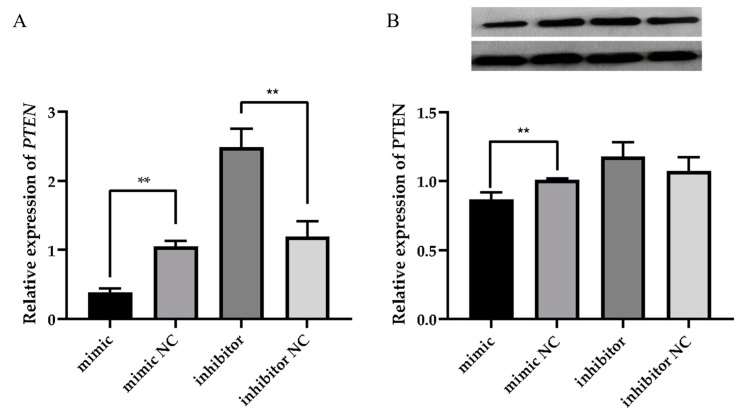
Effect of miR-30a-3p on PTEN expression in ovine MECs. (**A**) Relative expression level of mRNA. (**B**) Expression level of protein. The values are shown as mean ± SD (*n* = 3). ** *p* < 0.01.

The ovine MECs were transfected with miR-30a-3p mimic, miR-30a-3p inhibitor, and corresponding negative controls 48 h later. The RT-qPCR results showed that overexpression of miR-30a-3p significantly inhibited *PTEN* gene expression, whereas silencing of miR-30a-3p promoted *PTEN* gene expression (Figure 5A, *p* < 0.01). Western blot results showed that overexpression of miR-30a-3p also significantly inhibited PTEN protein expression, whereas silencing of miR-30a-3p had no effect on PTEN protein expression. (Figure 5B, *p* > 0.05).

**Figure 6:**
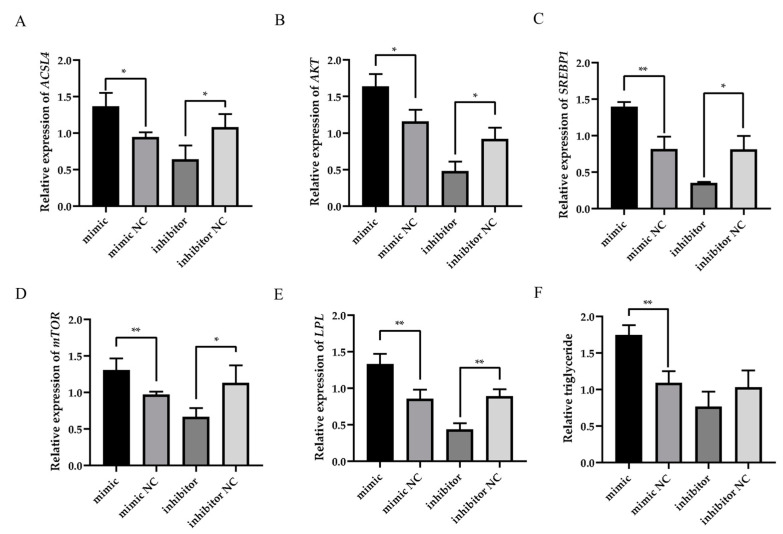
Effect of ACSL4 (**A**), AKT (**B**), SREBP1 (**C**), mTOR (**D**), LPL (**E**), and triglyceride level (**F**) on ovine MECs when the miR-30a-3p mimic, the miR-30a-3p inhibitor, and their NC were transfected into ovine MECs. The values are shown as mean ± SD (*n* = 3). ** *p* < 0.01 and * *p* < 0.05.

The RT-qPCR results demonstrated that miR-30a-3p overexpression significantly upregulated the mRNA levels of milk fat synthesis-related genes (*ACSL4*, *SREBP1*, *mTOR*, and *LPL*) in ovine MECs (*p* < 0.01). Meanwhile, it also significantly increased the expression of *AKT* (*p* < 0.05). In contrast, miR-30a-3p silencing significantly downregulated the expression of *ACSL4*, *AKT*, *SREBP1*, and *mTOR* (Figure 6, *p* < 0.05). It also significantly downregulated the expression of *LPL* (*p* < 0.01). Triglyceride quantification further revealed that miR-30a-3p overexpression significantly increased triglyceride accumulation in ovine MECs (*p* < 0.01), whereas miR-30a-3p silencing showed no statistically significant effect on triglyceride levels compared to negative controls (Figure 6F, *p* > 0.05). These findings collectively suggest that miR-30a-3p positively regulates triglyceride biosynthesis in ovine MECs.

## 4. Discussion

### 4.1. miR-30a-3p Expression Exhibits Tissue-Specific and Spatiotemporal Specificity

This study revealed that miR-30a-3p exhibits spatiotemporal and tissue-specific expression patterns, a phenomenon well reported for other miRNAs such as miR-200c [19], miR-148a [29], miR-26a/b [30], and miR-205 [31]. These observations highlight the complexity of miRNA-mediated gene regulation, where individual miRNAs can target different genes in different tissues to orchestrate different biological processes. It is estimated that miRNAs regulate over 60% of protein-coding genes in mammals [32]. These results suggest that miRNAs also have important regulatory roles for mammary gland development and lactation. This finding is consistent with our previous observation in small-tailed Han sheep, where miR-30a-3p expression was three times higher during peak lactation than in non-pregnant periods [24]. Notably, miR-30a-3p expression in the mammary gland exhibited dynamic changes across lactation stages, with the highest levels observed in pre-lactation, suggesting a potential correlation with milk production capacity. This is consistent with reports that milk yield and nutrient density are typically increased during early lactation; they found that with the birth of the lambs, the same change occurred in the lactation of the ewe [33]. Furthermore, miR-30a-3p was shown to be downregulated during mammary gland involution in mice compared to active lactation [25], reinforcing its functional relevance to lactogenesis.

We also observed the expression characteristics of miR-30a-3p in different organ tissues, which may reflect its functional diversity as a miRNA. Specifically, miR-30a-3p showed high expression in the lung, spleen, muscle, and mammary gland, suggesting tissue-specific regulatory roles. For example, given the importance of the lung and spleen in immune responses, elevated miR-30a-3p levels in these organs may fine-tune inflammatory and immunoregulatory pathways, consistent with previous reports [34]. Notably, miR-30a-3p is recognized as a key regulator of cancer cell invasion and proliferation. In lung adenocarcinoma, it inhibits tumor growth by targeting the GOLM1/JAK-STAT pathway [35], while in myoblasts, it promotes differentiation by suppressing proliferation [36]. Similarly, its enrichment in muscle and mammary glands is consistent with a potential role in myogenesis and lactation regulation. In contrast to its high expression in immune and metabolic tissues, the low abundance of miR-30a-3p in the kidney suggests either a minimal functional role or strong tissue-specific repression [37]. These spatially divergent expression patterns underscore the importance of miRNA-mediated post-transcriptional control in tissue homeostasis and highlight miR-30a-3p as a candidate biomarker or therapeutic target for diseases affecting the lung, spleen, muscle, and mammary gland. However, further mechanistic studies are warranted to dissect its precise functional contributions in these tissues.

### 4.2. miR-30a-3p Promotes the Viability and Number of Ovine MECs

The ovine MECs exhibit specialized lactogenic capacity and represent physiologically relevant models for investigating molecular mechanisms underlying lactation in vitro [4]. Given the high expression of miR-30a-3p in the mammary gland, we further investigated its function in ovine MECs. To initially explore the function of miR-30a-3p in mammary gland development and lactation, we conducted experiments in which we overexpressed and silenced miR-30a-3p in ovine MECs. Our study revealed, through the application of CCK8 and Edu techniques, that overexpression of miR-30a-3p enhances the vitality and proliferation of ovine MECs, whereas its silencing exhibits the opposite effect. This finding aligns with the high expression of miR-30a-3p observed in pre-lactation mammary gland tissue, further supporting the hypothesis that miR-30a-3p may play a role in promoting the secretory ability of mammary epithelial cells. This study found that the number and activity of MECs serve as indirect indicators of the lactation ability of female livestock, a concept that has been validated in cows and goats [38]. Numerous miRNAs have also been identified to exert similar effects in cows, goats, and sheep [11,39]. For example, miR-200c has been shown to regulate cell proliferation and differentiation in the mammary gland of sheep, while miR-145 has been implicated in the regulation of lactation-related gene expression in cows [40]. Studies have shown that the same miRNA could target many different genes, and the same gene could be regulated by multiple miRNAs. The motifs formed by miRNAs and their target genes can not only regulate the growth and development of organisms but also play important roles in reproduction, metabolism, and disease [14,15]. However, the interactions between miRNAs need to be further investigated in the mammary glands of sheep.

### 4.3. PTEN Is a Direct Target Gene of miR-30a-3p

Research has also shown that miRNAs primarily exert post-transcriptional regulatory functions in cells [32,40]. By integrating predictions from miRanda 3.3a, miRDB, and TargetScan 8.0, we identified 86 common target genes of miR-30a-3p. KEGG enrichment analysis revealed that these genes are significantly associated with 22 pathways, including metabolic pathways, fatty acid biosynthesis/degradation, and key signaling cascades linked to mammary development and lactation, such as the PI3K-Akt, mTOR, focal adhesion, and insulin resistance signaling pathways. These pathways are implicated in diverse cellular processes, including immunity, metabolism, and growth [40]. Notably, PTEN emerged as a key candidate among the predicted targets due to its well-established role in mammary biology. As a tumor suppressor, PTEN encodes a phosphatase that antagonizes the PI3K/AKT pathway, a central regulator of cell proliferation and survival [41]. Accumulating evidence highlights PTEN’s critical function in mammary gland development, particularly in modulating milk protein synthesis and secretion. For instance, a study by Wang et al. (2014) demonstrated that *PTEN* suppresses lactation in dairy cows by downregulating β-casein, triglyceride, and lactose secretion in MECs of dairy cows, consistent with earlier findings on its regulatory role in lactogenic pathways [42,43]. Furthermore, conditional PTEN deletion in mouse mammary epithelial cells leads to differentiation defects and hyperproliferation, ultimately driving tumorigenesis with basal-like characteristics [44,45]. These collective findings position PTEN as a pivotal node in miR-30a-3p-mediated regulation of lactogenesis.

### 4.4. MiR-30a-3p Regulates Milk Fat Synthesis by Targeting PTEN in Ovine MECs

Studies have reported that miRNAs are involved in post-transcriptional regulation by degrading or repressing the expression of target genes [14,15]. The ovine MECs were transfected with miR-30a-3p mimic, miR-30a-3p inhibitor, and corresponding negative controls 48 h later. The RT-qPCR results showed that overexpression of miR-30a-3p significantly inhibited *PTEN* gene expression while silencing miR-30a-3p promoted *PTEN* gene expression. Meanwhile, the Western blot results showed that overexpression of miR-30a-3p significantly inhibited PTEN protein expression, while silencing miR-30a-3p promoted PTEN protein expression. This result suggests that *PTEN* is a direct target gene of miR-30a-3p, which can inhibit the expression of its target genes. *PTEN* has also been reported to be regulated by a variety of miRNAs in a wide range of tissues. For example, Zhang et al. (2016) found that miR-148a can target PTEN in MG63 cells and inhibit its expression [46]. Additionally, Jin et al. (2021) demonstrated that miR-148a regulates the proliferation and differentiation of ovine preadipocytes by targeting PTEN [47]. Studies have further shown that overexpression of *PTEN* may inhibit the adipocyte differentiation process and decrease the expression of milk fat synthesis genes such as *PPARγ*, *FASN*, *GLUT4*, *C/EBPβ*, *FATP4*, and *LPL* [47,48].

Our findings indicate a pivotal role for miR-30a-3p in regulating the expression of milk fat synthesis-related genes in ovine MECs. Specifically, the overexpression of miR-30a-3p was found to upregulate the expression levels of *ACSL4*, *AKT*, *SREBP1*, *mTOR*, and *LPL*, genes that are crucial for fatty acid activation, signaling pathways promoting lipid synthesis, sterol regulatory element binding, protein synthesis regulation, and lipoprotein lipase activity, respectively [49,50,51]. For example, a study found that zearalenone exposure downregulated the mRNA/protein levels of these genes involved in milk fat synthesis by disrupting the AKT-mTOR-PPARγ-ACSL4 pathway [52]. Conversely, the silencing of miR-30a-3p led to a significant downregulation of these marker genes, suggesting a direct correlation between miR-30a-3p expression and the activation of milk fat synthesis pathways. Triglyceride detection further demonstrated that miR-30a-3p overexpression enhanced triglyceride production, but miR-30a-3p silencing did not change triglyceride synthesis in ovine MECs. Triglycerides are the main component of milk fat, accounting for about 98–99%, with the remainder being phospholipids and a small amount of sterols, free fatty acids, and fat-soluble vitamins. It can, therefore, directly reflect the fat content of milk. These data demonstrate that miR-30a-3p greatly enhances milk fat metabolism in ovine MECs [49]. Unfortunately, more functions in the mammary gland or animal body need to be further investigated in animal models to reveal other functions of miR-30a-3p.

## 5. Conclusions

This study found that miR-30a-3p promoted the viability and number of ovine MECs. Meanwhile, miR-30a-3p affected the expression of marker genes related to mammary lipid synthesis by targeting *PTEN*, which, in turn, promoted triglyceride synthesis. This result provides a better understanding of the function of miR-30a-3p in MECs and provides an idea for a better understanding of the regulatory mechanisms of lactation.

## Data Availability

The data presented in this study are available in this article and the Supplementary Materials.

## Supplementary Materials

The following supporting information can be downloaded at https://www.mdpi.com/article/10.3390/ani15081180/s1, Table S1: Sequence information of primers designed for PCR and RT-qPCR.
