# Supplementary material for: MicroRNA-30a-3p Influences Milk Fat Metabolism by Targeting PTEN in Mammary Epithelial Cells of Sheep
Source: Animals (Basel). 2025 Apr 20;15(8):1180. doi: 10.3390/ani15081180 (PMC12023941; doi:10.3390/ani15081180)
Supplement: Supplementary file 1 [file animals-15-01180-s001.zip › animals-3550710-supplementary.pdf]

Table S1. Sequence information of primers designed for PCR and RT-qPCR

Name	**Primer**	**Sequence**	**Amlicon size (bp)**	**Gene ID**	**Purpose of primers**
PTEN (WT)	Forward	CCGCTCGAGGAAGGTCTGAATGAGGGTT	933	XM_042238662	pmiR-RB-Report™
	Reverse	GAATGCGGCCGCATCTGATTGGCAGGAGGC			
PTEN (MUT)	Forward	TGTGACTTTGGTTTCCGAGTCCTAATTAAAACTTT			
	Reverse	CGGAAACCAAAGTCACATAGCAATTCTTGTCAAATTCTATGG			
PTEN	Forward	ACGACGGGAAGACAAGTT	158		RT-qPCR
	Reverse	CCTCTGGTCCTGGTATGA			
ACSL4	Forward	TCTCCTTCTTCCCAAACT	138	XM_042242177.1	
	Reverse	AGCAGTGACACCGTTCATGAC			
LPL	Forward	ACCTGAAGACTCGTTCTC	206	NM_001009394.1	
	Reverse	CACCTCCGTGTAAAGTAG			
mTOR	Forward	AACAGCGAGCACAAGGAG	134	NM_001145455.1	
	Reverse	AGCACATCTGCCACCACT			
SREBP1	Forward	ACAGCCCGGTCTTTGAGG	200	XM_027974784.2	
	Reverse	CCCAGGACGGTGGTTGAT			
AKT	Forward	GCTCTTCTTCCACCTGTCCC	162	NM_001161857.1	
	Reverse	ATGTGCCCGTCCTTGTCC			
GAPDH	Forward	GTCGGAGTGAACGGATTT	174	NM_001190390.1	
	Reverse	CTCTGCCTTGACTGTGCC			
MiR-30a-3p	Forward	CTTTCAGTCGGATGTTTGCAG			
	Reverse	/			
U6	Forward	ACGGACAGGATTGACAGATT			
	Reverse	TCGCTCCACCAACTAAGAA			

Note：WT: The primers used for constructing wild-type (WT) pmiR-RB-Report™ vector.

MUT: The primers used for constructing mutated (MUT) pmiR-RB-Report™ vector.
